# Cyclophosphamide for interstitial lung disease-associated acute respiratory failure: mortality, clinical response and radiological characteristics

**DOI:** 10.1186/s12890-021-01615-2

**Published:** 2021-07-28

**Authors:** Johanna P. van Gemert, Inge A. H. van den Berk, Esther J. Nossent, Leo M. A. Heunks, Rene E. Jonkers, Alexander P. Vlaar, Peter I. Bonta

**Affiliations:** 1 Department of Intensive Care Medicine, Amsterdam University Medical Center, Location AMC, University of Amsterdam, Amsterdam, The Netherlands; 2 Department of Radiology, Amsterdam University Medical Center, Location AMC, University of Amsterdam, Amsterdam, The Netherlands; 3 Department of Respiratory Medicine, Amsterdam University Medical Center, Location VUMC, Vrije Universiteit, Amsterdam, The Netherlands; 4 Department of Intensive Care Medicine, Amsterdam University Medical Center, Location VUMC, Vrije Universiteit, Amsterdam, The Netherlands; 5 Department of Respiratory Medicine, Amsterdam University Medical Center, Location AMC, University of Amsterdam, Amsterdam, The Netherlands; 6 Department of Pulmonary Diseases, University Medical Center Groningen (UMCG), Hanzeplein 1, HP BB72, 9700 RB Groningen, The Netherlands

**Keywords:** Interstitial lung disease, Acute respiratory failure, Cyclophosphamide, Ground-glass opacification

## Abstract

**Background:**

Treatment for interstitial lung disease (ILD) patients with acute respiratory failure (ARF) is challenging, and literature to guide such treatment is scarce. The reported in-hospital mortality rates of ILD patients with ARF are high (62–66%). Cyclophosphamide is considered a second-line treatment in steroid-refractory ILD-associated ARF. The first aim of this study was to evaluate the in-hospital mortality in patients with ILD-associated ARF treated with cyclophosphamide. The second aim was to compare computed tomographic (CT) patterns and physiological and ventilator parameters between survivors and non-survivors.

**Methods:**

Retrospective analysis of patients with ILD-associated ARF treated with cyclophosphamide between February 2016 and October 2017. Patients were categorized into three subgroups: connective tissue disease (CTD)-associated ILD, other ILD or vasculitis. In-hospital mortality was evaluated in the whole cohort and in these subgroups. Clinical response was determined using physiological and ventilator parameters: Sequential Organ Failure Assessment Score (SOFA), PaO2/FiO2 (P/F) ratio and dynamic compliance (Cdyn) before and after cyclophosphamide treatment. The following CT features were quantified: ground-glass opacification (GGO) proportion, reticulation proportion, overall extent of parenchymal disease and fibrosis coarseness score.

**Results:**

Fifteen patients were included. The overall in-hospital mortality rate was 40%. In-hospital mortality rates for CTD-associated ILD, other ILD and vasculitis were 20, 57, and 33%, respectively. The GGO proportion (71% vs 45%) was higher in non-survivors. There were no significant differences in the SOFA score, P/F ratio or Cdyn between survivors and non-survivors. However, in survivors the P/F ratio increased from 129 to 220 mmHg and Cdyn from 75 to 92 mL/cmH2O 3 days after cyclophosphamide treatment. In non-survivors the P/F ratio hardly changed (113–114 mmHg) and Cdyn even decreased (27–20 mL/cmH2O).

**Conclusion:**

In this study, we found a mortality rate of 40% in patients treated with cyclophosphamide for ILD-associated ARF. Connective tissue disease-associated ILD and vasculitis were associated with a lower risk of death. In non-survivors, the CT GGO proportion was significantly higher. The P/F ratio and Cdyn in survivors increased after 3 days of cyclophosphamide treatment.

**Supplementary Information:**

The online version contains supplementary material available at 10.1186/s12890-021-01615-2.

## Background

Acute respiratory failure (ARF) is one of the most severe complications of interstitial lung disease (ILD). Patients with severe ARF require mechanical ventilation in the intensive care unit (ICU). The reported in-hospital mortality rates of ILD patients with ARF are high, and the outcomes of the different ILD subtypes are quite variable. For a mixed population of ILD patients with ARF, in-hospital mortality rates are as high as 66% [1]. The highest mortality rates in patients with ARF are reported for idiopathic pulmonary fibrosis (IPF) (73–100%), while mortality rates in connective tissue disease (CTD)-associated ILD (62%) and drug-induced ILDs (64%) are slightly lower [1–3]. Treatment of patients with ILD-associated ARF is particularly challenging. Intravenous corticosteroid therapy is the initial treatment of choice for ILD-associated ARF. According to British Thoracic Society (BTS) guidelines, cyclophosphamide is the second-line treatment in steroid-refractory ILD-associated ARF (evidence level D) [4]. For vasculitis, the combination of corticosteroid therapy and intravenous cyclophosphamide or rituximab is considered the first-line treatment [5]. However, for patients with ILD-associated ARF there are no randomized controlled trials available. Overall, the optimal treatment strategy to manage the heterogeneous groups of ILD patients with ARF in the ICU is not well established.

Radiologic evaluation through computed tomography (CT) is essential for the characterization and classification of ILD. Limited data are available on how parenchymal changes on CT scans can predict prognosis in ILD patients with severe respiratory failure in need of invasive ventilation.

In this study, we focused on the clinical response and radiological characteristics of mechanically ventilated patients who received cyclophosphamide for ILD-associated ARF. As ILDs are a very heterogeneous group of diseases with several different causes, patients were categorized into 3 subgroups: connective tissue disease (CTD)-associated ILD, other ILD (non-IPF) or vasculitis.

The first aim of this study was to evaluate in-hospital mortality in the whole cohort and in the 3 different subgroups. The second aim was to compare computed tomographic (CT) patterns and physiological and ventilator parameters between survivors and non-survivors.

## Material and methods

### Patients

Data were collected from adult patients with ILD-associated ARF who were mechanically ventilated and treated with cyclophosphamide at the ICUs of two tertiary teaching hospitals in the Netherlands between February 2016 and October 2017. All surviving patients provided written informed consent to the study. Data collection was performed in accordance with the institutional guidelines. ILD diagnosis was defined based on the American Thoracic Society/European Respiratory Society classification of idiopathic interstitial pneumonias and the EULAR/ERA-EDTA recommendations for the management of ANCA-associated vasculitis [5, 6]. The diagnosis was confirmed by formal multidisciplinary team discussion. All patients were treated with intravenous corticosteroids before receiving cyclophosphamide according to the BTS guidelines [4]. Steroid-refractory disease was defined as lack of clinical improvement or progressive respiratory failure despite adequate ventilation after corticosteroid treatment [4]. Patients were categorized into three subtypes: CTD-associated ILD, other ILD or vasculitis. Patients with IPF were not included in the study. IPF patients with ARF are generally not intubated or treated with cyclophosphamide in the participating hospitals. Demographic, diagnostic and clinical data were taken from the medical records. The primary end-point was in-hospital mortality. Secondary endpoints were computed tomographic (CT) patterns and clinical response. Clinical response was determined using the following physiological and ventilatory parameters: Sequential Organ Failure Assessment Score (SOFA), PaO2/FiO2 ratio (P/F ratio) and dynamic compliance (Cdyn) before and 3 days after cyclophosphamide treatment.

### Quantification of CT scan patterns

Two investigators (a subspecialty thoracic radiologist and a respiratory physician) retrospectively reviewed the CT scans at five levels: (1) origin of great vessels; (2) carina; (3) pulmonary venous confluence; (4) between levels (3) and (5); and (5) 1 cm above the right hemi-diaphragm. An imaging example of a CT scans at five levels can be found in Supplementary Figure 1.

The investigators were blinded to outcomes. The CT assessment was consensus based.

**Fig. 1:**
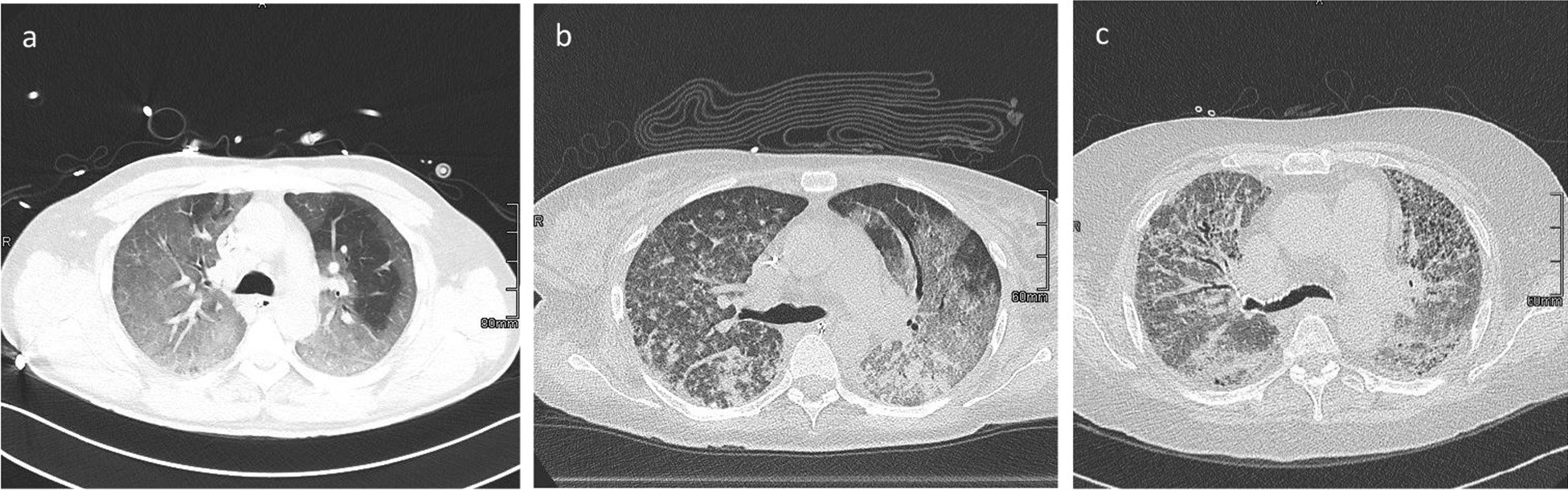
Examples of CT coarseness sore. **a** Coarseness score 1 (fine intralobular fibrosis); **b** coarseness score 2 (microcystic reticular pattern comprising air spaces smaller than or equal to 4 mm in diameter); **c** coarseness score 3 (macrocystic reticular pattern comprising air spaces larger than 4 mm in diameter)

Based on the study of Desai et al., the following features were quantified at each level: ground-glass opacification (GGO) proportion (GGO percentage of the total lung parenchyma), reticulation proportion (reticulation percentage of the total lung parenchyma), the overall extent of parenchymal lung disease (including GGO, reticulation and consolidation) and fibrosis coarseness score [7]. A reticular pattern was defined as interlobular septal thickening, intralobular interstitial thickening, wall cysts or honeycombing, peribronchovascular interstitial thickening and traction bronchiectasis/bronchiolectasis. The GGO pattern was defined as a hazy increase in opacity with preservation of bronchial and vascular markings. The coarseness score was determined as follows: (0) no fibrosis; (1) fine intralobular fibrosis; (2) microcystic reticular pattern comprising air spaces smaller than or equal to 4 mm in diameter; and (3) a macrocystic reticular pattern comprising air spaces larger than 4 mm in diameter. The overall coarseness score for each patient was calculated by summing the scores at five levels [7]. Imaging examples of the CT coarseness score can be found in Fig. 1.

### Statistical analysis

The Mann–Whitney U-test and chi-squared test were used to compare patient characteristics and CT features between survivors and non-survivors. Analyses were carried out using SPSS for Mac. A p value of less than 0.05 was used as the cut-off for significance.

## Results

Fifteen patients with ILD-associated ARF were admitted to the ICUs of the two university hospitals within the study period (shown in Table 1 and Supplementary Table 1). There were 5 patients (33%) with CTD-associated ILD, 7 (47%) with other ILD and 3 (20%) with vasculitis (shown in Table 2). Three patients (20%) had previously been diagnosed with ILD, while 12 (80%) were diagnosed with ILD upon admission. The median age was 56 years (IQR, 44–62), ten patients (67%) were male, and 8 (53%) were ever-smokers. The most frequent comorbidities reported in these patients were systemic autoimmune disease (53%), cardiovascular disease (27%), diabetes (20%) and hypertension (13%). Five out of 15 (33%) patients had a history of cancer (clinically undetectable and without active treatment during the study).

**Table 1:** Cases of interstitial lung disease associated acute respiratory failure

Case	Sex (f/m)	Age (years)	Smoking (Y\N)	Comorbidity/history	Diagnosis	CT findings	Pathology	BAL	Antibiotics	Therapy (cd)	In-hospital outcome, cause of death
1	F	62	Y	Dermato-myositis PMH: Breast cancer	Anti-MDA5 dermato-myositis	OP with architectural distortion	N/A	ng	Augmentin, ceftazidime^a^	MPS^b^ 1 g CYC^c^ 600 mg/m^2^	Death, progressive respiratory failure
2	M	60	N	UCTD AF, DM, OSAS	UCTD-ILD	OP with architectural distortion	N/A	ng	SDD	MPS 3 g CYC 600 mg/m^2^	Survival
3	F	60	N	AVNRT	Medium-size-artery vasculitis	Diffuse GGO and consolidation	Vasculitis DAH	ng	SDD	MPS 6 g CYC 750 mg/m^2^ IVIG	Survival
4	M	41	N	GPA	GPA	Diffuse GGO	Eosinophilic necrotizing granulomatous inflammation	ng	SDD	MPS 3 g Continuous oral CYC 2 mg/kg Plasmapheresis	Survival
5	F	40	N	Neuro-fibromatosis type 1 RA	ANCA-negative small vessel vasculitis	Diffuse GGO	Vasculitis DAH	ng	SDD	MPS 12 g CYC 750 mg/m^2^ IVIG Plasmapheresis	Death MOF
6	M	61	N	None	Methotrexate-induced lung injury	Fibrotic NSIP	N/A	ng	SDD moxifloxacin^a^	MPS 3 g CYC 600 mg/m^2^	Survival
7	M	55	Y	Alcoholism, coronary heart disease	AIP	Diffuse GGO with architectural distorsion	N/A	ng	Augmentin Doxycycline^a^	MPS 3 g CYC 500 mg/m^2^ ECLA	Death Progressive respiratory failure
8	M	44	Y	SLE	SLE-related ILD	NSIP	N/A	CMV	Ganciclovir SDD	MPS 3 g CYC 600 mg/m^2^ Plasmapheresis	Survival
9	M	72	Y	Poliomyelitis PMH: esophageal carcinoma	AIP	Diffuse GGO with architectural distorsion	N/A	ng	Oseltamivir Ceftriaxone Erythromycine^a^	MPS 3 g CYC 600 mg/m^2^	Survival
10	M	52	N	MCTD PMH: NHL	MCTD-ILD	NSIP	NA	S.aureus	SDD flucloxacillin	MPS 3 g CYC 500 mg/m^2^	Survival
11	F	38	N	SLE	SLE	Diffuse GGO	N/A	ng	Ceftriaxone Azithromycin^a^	MPS 3 g Plasmapheresis CYC 750 mg/m^2^	Survival
12	F	68	Y	PMH: breast cancer and esophageal carcinoma	Unclassified ILD	Unclassified ILD with signs of fibrosis	Fibrosis with organization	ng	SDD	MPS 6 g CYC 600 mg/m^2^	Death Progressive respiratory failure
13	M	71	Y	AF, HPT, RA	AIP	Diffuse GGO, with architectural distorsion	DAD	ng	Ceftriaxone Ciprofloxacin^a^	MPS 3 g CYC 750 mg/m^2^	Survival
14	M	54	Y	DM PMH: Hodgkin lymphoma	EAA	Diffuse GGO	N/A	ng	SDD	MPS 3 g CYC 600 mg/m^2^	Death Blocked tracheostomy Progressive respiratory failure
15	M	56	Y	DM, HPT, COPD	Unclassified ILD	Unclassified ILD with signs of fibrosis	DAD	ng	Augmentin Ciprofloxacin^a^	MPS 3 g CYC 600 mg/m^2^	Death

AF, atrial fibrillation; AIP, acute interstitial pneumonia; ANCA, anti-neutrophil cytoplasmic antibody; AVNRT, atrioventricular nodal reentry tachycardia; COPD, chronic obstructive pulmonary disease; cd, cumulative dose; CT, computed tomography; CYC, cyclophosphamide; DAD, diffuse alveolar damage; DAH, diffuse alveolar hemorrhage; DM, diabetes mellitus; EAA, extrinsic allergic alveolitis; ECLA, extra corporeal lung assist; GGO, ground glass opacity; GPA, granulomatosis with polyangiitis; HPT, hypertension; ICU, intensive care unit; ILD, interstitial lung disease; IVIG, intravenous immunoglobulin; LOS, length of stay; MCTD, mixed connective tissue disease; MOF, multiple organ failure; MPS, methylprednisolone; N/A, not applicable; ng, no growth; NHL, non-Hodgkin lymphoma; NSIP, non-specific interstitial pneumonia; OP, organizing pneumonia; OSAS, obstructive sleep apnoea syndrome; PMH, past medical history; RA, rheumatoid arthritis; SDD, selective digestive decontamination; SLE, systemic lupus erythematosus; UCTD-ILD, undifferentiated connective tissue disease associated interstitial lung disease

^a^Empirical antibiotic therapy

^b^MPS 1000 mg/day for 3 days

^c^Cyclophosphamide single dose

**Table 2:** In-hospital mortality of interstitial lung disease patients with acute respiratory failure treated with cyclophosphamide

	All	Other ILD	CTD-ILD	Vasculitis
n	15	7	5	3
Deaths, no	6	4	1	1
Mortality (%)	40	57	20	33

CTD-ILD, connective tissue disease-associated interstitial lung disease; ILD, interstitial lung disease

All patients had severe, steroid-refractory ARF requiring invasive mechanical ventilation and received cyclophosphamide in addition to standard care, including empirical antibiotic therapy or selective digestive decontamination (SDD) and (methyl)prednisolone (shown in Table 1). According to ILD guidelines 1000 mg methylprednisolone was given for 3 days [4]. To exclude respiratory infections (bacterial, fungal, viral), bronchoscopy with bronchoalveolar lavage (BAL) was performed in all patients prior to the administration of methylprednisolone and cyclophosphamide. The median time between the administration of methylprednisolone and cyclophosphamide was 7 days (IQR 3–10). Only two patients (13%) had positive BAL culture results before the initiation of cyclophosphamide therapy (shown in Table 1). Before cyclophosphamide therapy, the median number of leukocytes was 13.8 × 10^9^/L (IQR 10.1–20.5).

Four patients (27%) received 750 mg/m^2^ cyclophosphamide; 8 patients (53%) received 600 mg/m^2^; 2 patients (13%) received 500 mg/m^2^ (dose adjustments because of impaired renal function); and 1 patient (7%) received a daily oral dose of 2 mg/kg. Leukopenia (defined as leukocytes < 4 × 10^9^/L) occurred in 2 patients after cyclophosphamide therapy and lasted for 5–9 days. Central line-associated bloodstream infection (n = 2; 13%) and ventilator-associated pneumonia (n = 2; 13%) were the most common infections, followed by urinary tract infection (n = 1; 6.7%). No life-threatening side effects were observed.

Additionally, two patients (13%) were treated with intravenous immunoglobulin, 4 patients (27%) underwent plasmapheresis, and one patient (7%) received extracorporeal lung assist (ECLA) support. The overall in-hospital mortality rate in all patients was 40% (shown in Table 2). In-hospital mortality rates for CTD-associated ILD, other ILDs and vasculitis were 20%, 57% and 33%, respectively (shown in Table 2). The median ICU length of stay (LOS) was 18 days (IQR 14–28). The median time to respond (extubation or successful weaning course) to cyclophosphamide was 9 days (IQR 6–14).

No statistically significant differences between survivors and non-survivors were found with respect to age, sex, smoking status or ILD subtype. The median time between ICU admission and cyclophosphamide administration was 10 days (IQR 5–14) in survivors and 9 days (IQR 8–15) in non-survivors (*p* = 0,813).

**Fig. 2:**
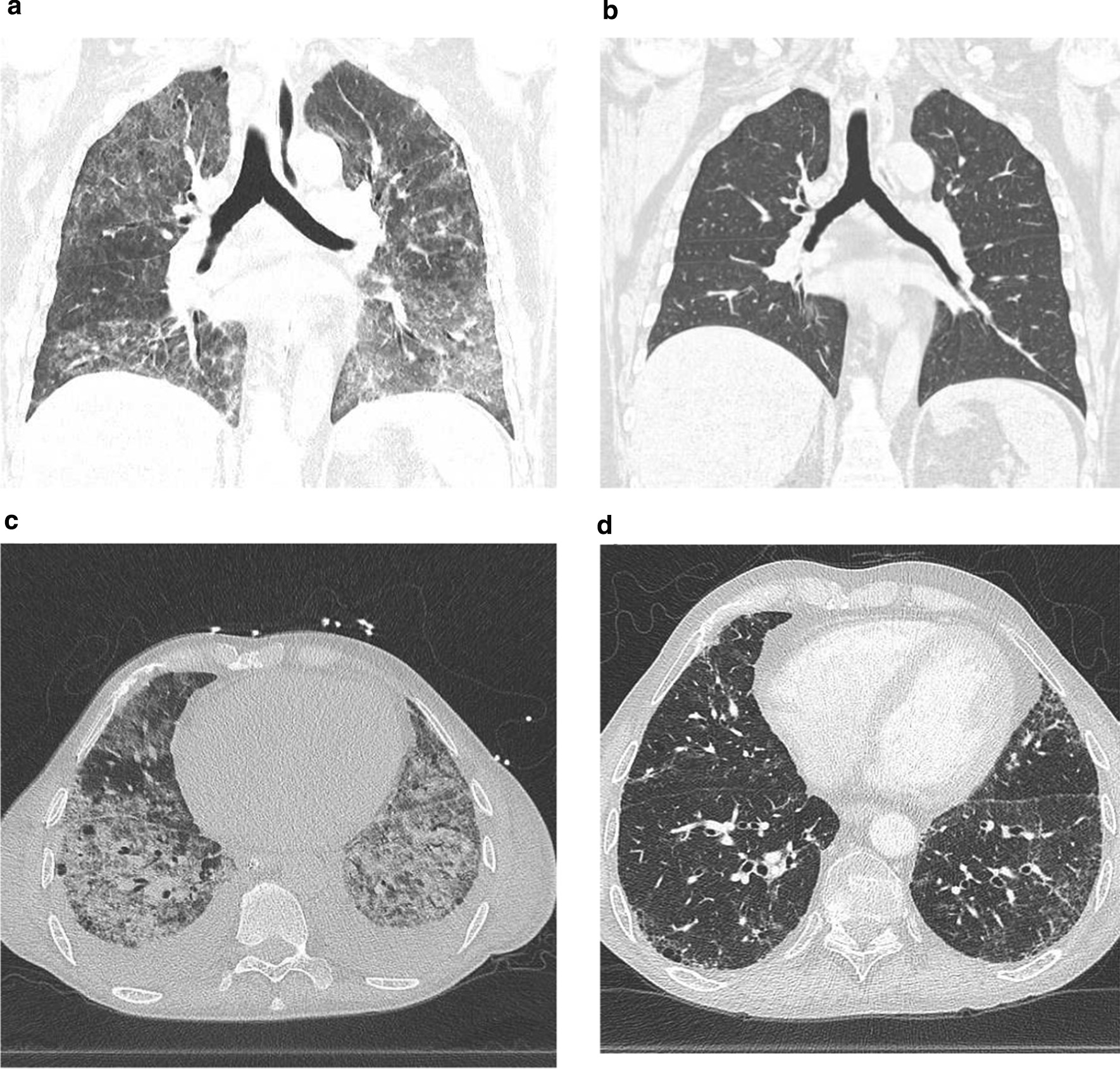
CT scans of two patients before cyclophosphamide treatment and after cyclophosphamide treatment. Coronal CT scan: case 8 (SLE-like disease) before cyclophosphamide treatment (**a**) and after cyclophosphamide treatment (**b**). Axial CT scan: case 10 (MCTD-ILD) before cyclophosphamide treatment (**c**) and after cyclophosphamide treatment (**d**)

The extent and severity of CT features in survivors and non-survivors are shown in Table 3. The GGO proportion score was significantly higher in non-survivors (71%; IQR 52–82) than in survivors (45%; IQR 30–56) (*p* = 0.034). In line with the CT-detected reticulation proportion, the fibrosis coarseness score was higher in non-survivors (7; IQR 4–10) than in survivors (1; IQR 0–5) but did not reach statistical significance (*p* = 0.066). Representative CT images before and after cyclophosphamide administration in 2 cases are shown in Fig. 2.

**Table 3:** Extent and severity of CT patterns of interstitial lung disease patients with acute respiratory failure treated with cyclophosphamide

CT pattern	Survivors (n = 9)	Non survivors (n = 6)	*p* value
Ground-glass opacification proportion (IQR)	45 (30–56)	71 (52–82)	0.034
Reticulation proportion (IQR)	8 (0–22)	26 (8–45)	0.150
Overall extent of parenchymal lung disease (IQR)	84 (63–92)	88 (75–91)	0.906
Coarseness score, median (IQR)	1 (0–5)	7 (4–10)	0.066

CT, computed tomograph; IQR, interquartile range

**Fig. 3:**
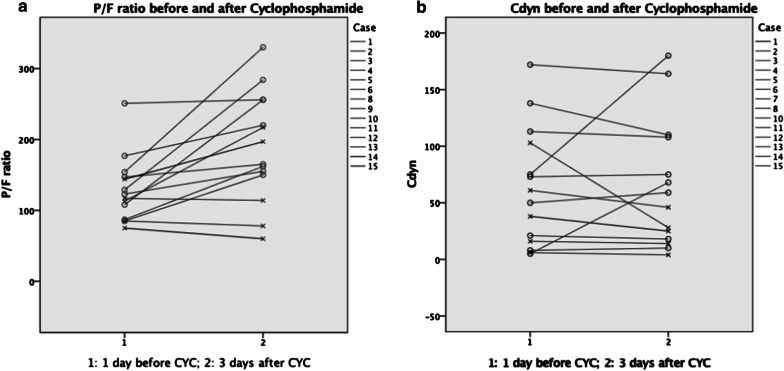
P/F ratio and dynamic compliance before and after cyclophosphamide therapy. o survivors; x non-survivors. **a** Case 7 was not shown: ECMO, therefore no realistic P/F ratio. **b** Case 9 was not shown: patient was extubated after cyclophosphamide treatment; therefore, Cdyn after cyclophosphamide was not available. cdyn: dynamic compliance; CYC: cyclophosphamide; P/F ratio: PaO2/FiO2-ratio

Additional physiological parameters and ventilator settings are shown in Table 4, Fig. 3 and Supplementary Table 1. There were no significant differences in the median SOFA score, P/F ratio or dynamic compliance (Cdyn) among survivors and non-survivors (Table 4). The median P/F ratio increased from 129 to 220 mmHg in survivors and from 113 to 114 mmHg in non-survivors 3 days after cyclophosphamide treatment. Median Cdyn increased from 75 to 92 mL/cmH2O in survivors and decreased from 27 to 20 mL/cmH2O in non-survivors 3 days after cyclophosphamide treatment.

**Table 4:** Physiological and ventilator parameters in survivors and non-survivors

	Survivors	Non-survivors	*p* value
SOFA	8 (5–13)	11 (8–13)	0.241
P/F ratio 1 day before MPS^a^ (mmHg)	156 (140–187)	122 (83–153)	0.119
P/F ratio 3 days after MPS (mmHg)	182 (156–221)	113 (99–187)	0.091
P/F ratio 1 day before CYC (mmHg)	129 (98–166)	113 (80–131)	0.214
P/F ratio 3 days after CYC (mmHg)	220 (159–270)	114 (69–207)	0.079
Cdyn 1 day before MPS (mL/cmH2O)	59 (34–87)	20 (17–42)	0.111
Cdyn 3 days after MPS (mL/cmH2O)	63 (51–91)	27 (14–61)	0.077
Cdyn 1 day before CYC (mL/cmH2O)	75 (41–126)	27 (8–63)	0.442
Cdyn 3 days after CYC (mL/cmH2O)	92 (52–151)	20 (9–36)	0.063

Values are expressed as median and interquartile range (IQR)

CYC, cyclophosphamide; Cdyn, dynamic compliance; MPS, methylprednisolone; P/F ratio, PaO2/FiO2-ratio; SOFA, Sequential Organ Failure Assessment;

^a^Methylprednisolone 1 g/day for 3 days

## Discussion

In this study, we report the in-hospital mortality, clinical response and radiologic characteristics of cyclophosphamide-treated mechanically ventilated patients with ILD-associated ARF. We found an overall in-hospital mortality rate of 40%. This is in line with the recent case series of Schulze et al. on 14 patients with ILD-associated ARF treated with cyclophosphamide. In this study, cyclophosphamide therapy was associated with a favourable outcome. However, only the minority of patients had non-vasculitis ILD [8]. Compared to previous reports on mixed ILD-associated ARF not treated with cyclophosphamide, we found lower mortality rates (40% vs 62–67%) [1–3]. A systematic review, including 50 studies on patients admitted to the intensive care unit (ICU) with ILD-associated ARF, showed overall in-hospital mortality rates in mechanically ventilated patients of 67% in mixed-ILD and 77% in IPF [9]. Consistent with previous studies, we found that CTD-associated ILD and vasculitis were associated with a lower risk of death than other ILDs (20% and 33% vs 57%) [1, 2, 5]. For life-threatening vasculitis, the following treatment strategies are well known and in accordance with current guidelines: glucocorticoids and either cyclophosphamide or rituximab [5]. Reported ICU survival rates amongst patients with vasculitis-associated ARF range from 25 to 50% [10]. For non-vasculitis, ILD data are scarce, and given the poor prognosis, add-on treatments are needed. Our findings might contribute to the awareness of cyclophosphamide as a treatment option for patients with ARF requiring ICU care, particularly in patients with vasculitis-associated ILD and MCTD-ILD.

Patients with IPF were not included in the study since in the participating hospitals, patients with IPF are rarely admitted to the ICU and generally treated with intravenous corticosteroids alone.

Radiologic evaluation through computed tomography (CT) is essential for the characterization and classification of ILD. In a retrospective analysis of 160 patients with ILD admitted to an ICU, analysis of the 76 with CT scans revealed a higher percent of fibrosing ILD among those who did not survive their ICU stay [11]. However, only 12 of the 160 patients were intubated. The CT scan analysis in our study might be used to predict the prognosis in ILD patients with more severe respiratory failure requiring invasive ventilation. Nevertheless, a larger confirmatory study is needed.

One of the most compelling observations of this study is the significantly higher ground-glass opacification (GGO) proportion in non-survivors than in survivors (71% vs 45%, respectively). The greater proportion of GGOs might be associated with more widespread impairment of alveolar and interstitial space and more prominent reduction of the surface for gas exchange. Therefore, the amount of GGO may be useful in predicting severe disease and the need for aggressive therapy. Furthermore, non-survivors had higher reticulation and fibrosis coarseness scores (26 vs 8; 7 vs 1, respectively). This might imply that a poor prognosis in ILD-associated ARF is associated with the initiation of the fibrotic process. The overall extent of disease may not be that significant, since it is partly determined by consolidation, which may be reversible.

The P/F ratio might be used to predict mortality in ARDS [12]. In our study, non-survivors have overall lower P/F ratio and Cdyn levels at baseline and 3 days after cyclophosphamide treatment. Given the small sample size, this difference did not reach statistical significance. There was a marked increase in the P/F ratio and Cdyn in survivors after 3 days of cyclophosphamide therapy. In non-survivors, the P/F ratio remained virtually unchanged, and Cdyn actually decreased (Table 3, Fig. 2). As such the P/F ratio and Cdyn could potentially be used to monitor the response to cyclophosphamide. Although P/F ratio and Cdyn seem to improve after methylprednisolone initially (Table 3), these patients were not able to wean from the ventilator or make further improvements after steroid therapy. Furthermore, the median time to respond to cyclophosphamide, defined as extubation or a successful weaning course, was 9 days. This may be useful for clinicians seeking to estimate time to treatment effect.

Cyclophosphamide is a highly potent immunosuppressant that comes with potential toxicities, including haemorrhagic cystitis, bone marrow suppression, increased risk of opportunistic infections, and malignancies [13]. However, the level of toxicity is related to the cumulative dose. In this study, only one cycle of cyclophosphamide was given. No severe adverse events were observed. Given the poor prognosis of ILD-associated ARF, the adverse event profile of cyclophosphamide is considered acceptable [10].

There are a number of limitations to our study that should be considered when interpreting our findings. First, as our study did not include a control group of untreated patients. Therefore, we cannot determine the impact of cyclophosphamide treatment on mortality in ILD-associated ARF. However, in our study the mortality of patients with ILD related ARF treated with cyclophosphamide was lower than the mortality in historical reports in patients not treated with cyclophosphamide. Second, the small size of our series limits the reliability of the results. Unfortunately, the low prevalence of ILD-associated ARF limits patient studies and data collection. Future studies in larger populations, each including a control group, are needed to confirm our findings.

## Conclusion

In this retrospective study, we found a mortality rate of 40% in patients treated with cyclophosphamide for ILD-associated ARF. Connective tissue disease-associated ILD and vasculitis ILD were associated with a lower risk of death than other ILDs when treated with cyclophosphamide. Furthermore, between survivors and non-survivors, we found that the GGO proportion was significantly higher in non-survivors. The P/F ratio and Cdyn in survivors increased after 3 days of cyclophosphamide treatment.

## Supplementary Information

**Additional file 1. Supplementary Table 1:** Physiological parameters and ventilator settings. *prone ventilation; **extracorporeal lung assist. Cdyn: dynamic compliance; CYC: cyclophosphamide; ICU LOS: Intensive Care Unit length of stay; MPS: methylprednisolone; n/a: Not Applicable; PEEP: positive end-expiratory pressure; P/F ratio: PaO2/FiO2-ratio; SOFA: Sequential Organ Failure Assessment; VT tidal volume

**Additional file 2. Supplementary Figure 1: **Imaging example of a CT scans at five levels. Level (1) origin of great vessels; level (2) carina; level (3) pulmonary venous confluence; level (4) between levels (3) and (5); and level (5) 1 cm above the right hemi-diaphragm

## Data Availability

The datasets used and/or analysed during the current study are available from the corresponding author on reasonable request.

## Abbreviations

AIP: Acute interstitial pneumonia
BAL: Bronchoalveolar lavage
ARF: Acute respiratory failure
BTS: British Thoracic Society
Cdyn: Dynamic compliance
CT: Computed tomography
CTD: Connective tissue disease
EAA: Extrinsic allergic alveolitis
ECLA: Extracorporeal lung assist
GGO: Ground-glass opacification
ICU: Intensive care unit
ILD: Interstitial lung disease
IMV: Invasive mechanical ventilation
IPF: Idiopathic pulmonary fibrosis
LOS: Length of stay
P/F ratio: PaO2/FiO2 ratio
SDD: Selective digestive decontamination
SOFA: Sequential Organ Failure Assessment Score
