# Supplementary material for: Cyclophosphamide for interstitial lung disease-associated acute respiratory failure: mortality, clinical response and radiological characteristics
Source: BMC Pulm Med. 2021 Jul 28;21:249. doi: 10.1186/s12890-021-01615-2 (PMC8316896; doi:10.1186/s12890-021-01615-2)
Supplement: Supplementary file 1 — Additional file 1. Supplementary Table 1: Physiological parameters and ventilator settings. *prone ventilation; **extracorporeal lung assist. Cdyn: dynamic compliance; CYC: cyclophosphamide; ICU LOS: Intensive Care Unit length of stay; MPS: methylprednisolone; n/a: Not Applicable; PEEP: positive end-expiratory pressure; P/F ratio: PaO2/FiO2-ratio; SOFA: Sequential Organ Failure Assessment; VT tidal volume [file 12890_2021_1615_MOESM1_ESM.docx]

| **Supplementary Table 1** | | | | | | | | | | | | | | | |
| --- | --- | --- | --- | --- | --- | --- | --- | --- | --- | --- | --- | --- | --- | --- | --- |
| Case | 1 | 2 | 3 | 4 | 5 | 6 | 7 | 8 | 9 | 10 | 11 | 12 | 13 | 14 | 15 |
| Survival (y/n) | n | y | y | y | n | y | n | y | y | y | y | n | y | n | n |
| SOFA | 8 | 4 | 6 | 3 | 11 | 8 | 14 | 8 | 7 | 14 | 13 | 12 | 12 | 7 | 11 |
| Leukocytes before CYC (x 10^9/L) | 20.5 | 13.8 | 10.1 | 15 | 5.2 | 17.9 | 25.4 | 30 | 18 | 8.5 | 6.4 | 12.3 | 22 | 11.4 | 13.2 |
| Time between MPS and CYC (days) | 7 | 2 | 3 | 3 | 4 | 7 | 13 | 2 | 13 | 10 | 5 | 10 | 7 | 10 | 7 |
| Time to respond to CYC (days) | died | 10 | 8 | 5 | died | 15 | died | 7 | 3 | 25 | 9 | died | 7 | 16 | died |
| ICU LOS | 8 | 27 | 38 | 6 | 45 | 15 | 14 | 21 | 14 | 40 | 18 | 28 | 17 | 25 | 9 |
| 1 day before MPS |  | | | | | | | | | | | | | | |
| P/F ratio | 98 | 132* | 180 | 164 | 175 | 150 | ** | 149 | 184 | 156 | 189 | 130 | 79 | 68 | 122 |
| VT (ml) | 360 | 570 | 340 | 540 | 405 | 600 | 200 | 780 | 460 | 700 | 570 | 530 | 470 | 15LO2 | 600 |
| PEEP (cm H2O) | 5 | 15 | 6 | 12 | 8 | 6 | 15 | 8 | 8 | 12 | 17 | 8 | 15 | n/a | 16 |
| Cdyn (mL/cmH2O) | 21 | 114 | 17 | 60 | 16 | 150 | 18 | 60 | 46 | 54 | 21 | 20 | 59 | n/a | 62 |
| 3 days after MPS |  | | | | | | | | | | | | | | |
| P/F ratio | 95 | 164* | 265 | 236 | 113 | 127 | ** | 206 | 158 | 182 | 154 | 207 | 203* | 167 | 102 |
| VT (ml) | 420 | 870 | 310 | 630 | 400 | 660 | 120 | 870 | 720 | 540 | 400 | 560 | 380 | 600 | 690 |
| PEEP (cm H2O) | 8 | 12 | 5 | 8 | 8 | 8 | 12 | 8 | 5 | 6 | 15 | 7 | 12 | 10 | 14 |
| Cdyn (mL/cmH2O) | 55 | 108 | 16 | 61 | 16 | 57 | 7 | 73 | 111 | 45 | 67 | 18 | 63 | 35 | 77 |
| 1 day before CYC |  | | | | | | | | | | | | | | |
| P/F ratio | 85 | 123* | 251 | 87 | 113 | 85 | ** | 108 | 154 | 177 | 147 | 117 | 129* | 144 | 75 |
| VT (ml) | 320 | 580 | 390 | 600 | 400 | 560 | 80 | 900 | 600 | 690 | 450 | 280 | 425 | 450 | 620 |
| PEEP (cm H2O) | 10 | 12 | 5 | 8 | 8 | 8 | 12 | 8 | 5 | 12 | 16 | 10 | 12 | 8 | 12 |
| Cdyn (mL/cmH2O) | 38 | 73 | 21 | 172 | 16 | 5 | 6 | 75 | 92 | 138 | 113 | 8 | 61 | 50 | 103 |
| 3 days after CYC |  | | | | | | | | | | | | | | |
| P/F ratio | 78 | 155* | 256 | 162 | 217 | 150 | ** | 256 | 330 | 220 | 165 | 114 | 184 | 197 | 60 |
| VT (ml) | 450 | 820 | 410 | 655 | 300 | 702 | 50 | 900 | 4 L O2 | 660 | 430 | 300 | 550 | 470 | 560 |
| PEEP (cm H2O) | 9 | 12 | 6 | 7 | 6 | 9 | 12 | 7 | n/a | 12 | 10 | 5 | 4 | 8 | 14 |
| Cdyn (mL/cmH2O) | 25 | 75 | 18 | 164 | 14 | 68 | 4 | 180 | n/a | 110 | 108 | 10 | 46 | 59 | 28 |
| Supplementary Table 1: Physiological parameters and ventilator settings  * prone ventilation; ** extracorporeal lung assist  Cdyn: dynamic compliance; CYC: cyclophosphamide; ICU LOS: Intensive Care Unit length of stay; MPS: methylprednisolone; n/a: Not Applicable; PEEP: positive end-expiratory pressure; P/F ratio: PaO2/FiO2-ratio; SOFA: Sequential Organ Failure Assessment; VT tidal volume | | | | | | | | | | | | | | | |
