# Supplementary material for: Cyclophosphamide for interstitial lung disease-associated acute respiratory failure: mortality, clinical response and radiological characteristics
Source: BMC Pulm Med. 2021 Jul 28;21:249. doi: 10.1186/s12890-021-01615-2 (PMC8316896; doi:10.1186/s12890-021-01615-2)
Supplement: Supplementary file 2 — Additional file 2. Supplementary Figure 1: Imaging example of a CT scans at five levels. Level (1) origin of great vessels; level (2) carina; level (3) pulmonary venous confluence; level (4) between levels (3) and (5); and level (5) 1 cm above the right hemi-diaphragm [file 12890_2021_1615_MOESM2_ESM.docx]

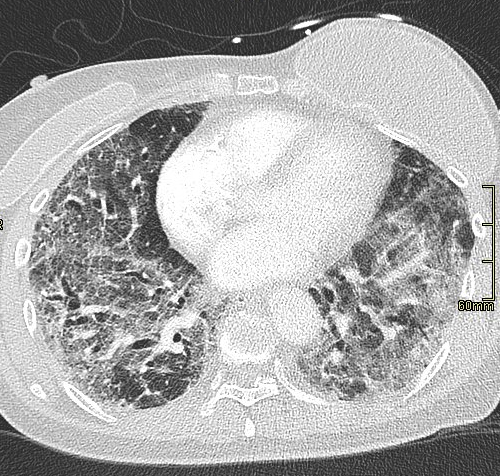

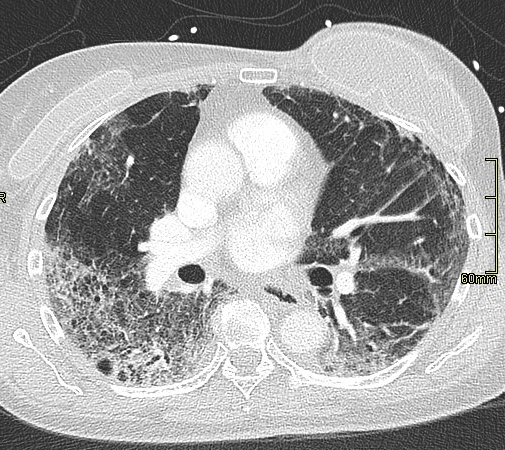

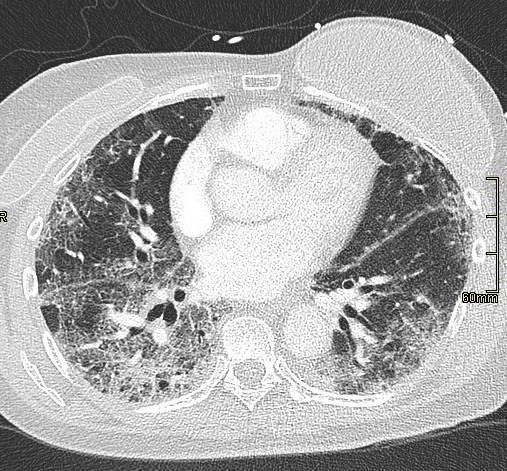

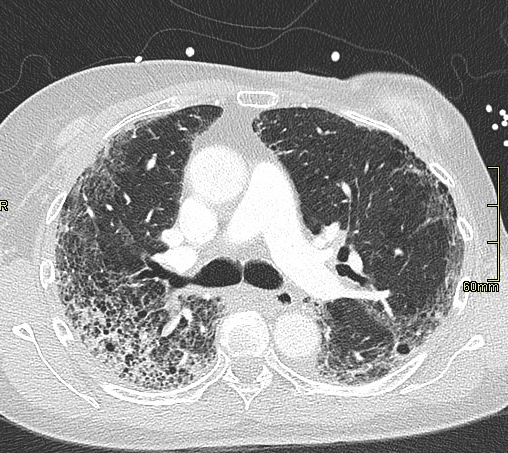

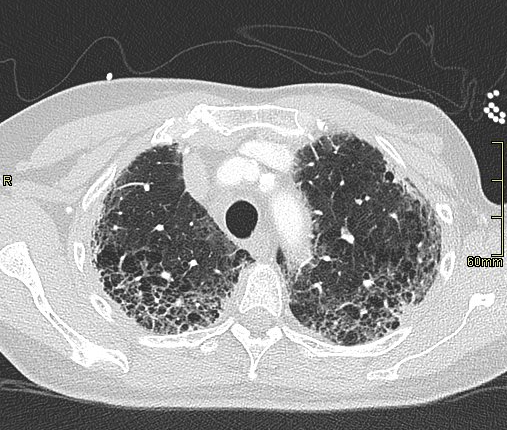


Supplementary Figure 1: Imaging example of a CT scans at five levels

Level (1) origin of great vessels; level (2) carina; level (3) pulmonary venous confluence; level (4) between levels (3) and (5); and level (5) 1 cm above the right hemi-diaphragm

Level 5

Level 4

Level 3

Level 2

Level 1

**Supplementary Figure 1**
